# Assessment of facultative anaerobes from the root canals of deciduous molars: An in vivo study

**DOI:** 10.15171/joddd.2017.018

**Published:** 2017-06-21

**Authors:** Manisha Chandwani, Shweta Chandak

**Affiliations:** ^1^Department of Pedodontics and Preventive Dentistry, Swargiya Dadasaheb Kalmegh Smruti Dental College and Hospital, Nagpur, Maharashtra, India

**Keywords:** Deciduous tooth, microorganisms, root canal

## Abstract

***Background.*** The current research aimed to assess the prevalence of facultative anaerobes isolated from the root canals of deciduous molars.

***Methods.*** The present research enrolled 60 children in the 6‒9-year age group based on clinical and radiographic findings. Under aseptic conditions, access cavities were prepared followed by collection of samples from infected root canals with the help of sterile paper points. The samples thus obtained were subjected for microbial assay.

***Results.*** It was found that *Enterococcus faecalis* was isolated in 30% of cases, *Escherichia coli* in 28.4%, *Staphylococcus aureus * in 25%, α-hemolytic *Streptococci* in 15% and *Proteus mirabilis* in 1.6% of cases.

***Conclusion.*** It was concluded from the results of the present study that the root canals of deciduous molars had predominance of facultative anaerobes, confirming its polymicrobial nature. This identification of microbes is crucial as it aids in understanding the pathogenesis of pulpal and periradicular diseases to provide effective antimicrobial irrigation and medicament for endodontic treatment.

## Introduction

Integrity of the deciduous dentition has to be maintained until normal exfoliation for proper development and maturation of the child, growth of the facioskeletal complex to its normal occlusion along with good esthetic qualities.^1^ Pediatric endodontics is more exasperating due to its root canal anatomy, proximity to the permanent tooth bud along with the difficulty encountered during management of the child’s behavior.^1-3^

Numerous factors contribute to the development of pulp and periapical diseases, eventually creating an opportunity for the ingress of bacteria in root canals. With the advent of strict anaerobic culture techniques, their sprouted a paradigm shift in the isolation of microorganisms from aerobes to facultative and obligate anaerobes from the permanent teeth with necrotic pulp and periapical pathosis.^4,5^

Currently, insignificant data is available regarding the identification of microbiota in deciduous teeth with pulp necrosis and periapical pathosis. However, documentation exists in relation to the polymicrobial nature of microbes predominated by anaerobes in the root canals of deciduous teeth with necrotic pulp and periapical lesions similar to permanent teeth.^6,7^ Various microorganisms isolated from infected root canals include *Streptococci*, *Staphylococcus*, *Diptheroids*, *Peptostreptococcus*, *Lactobacilli*, *Propionibacterium*, *Actinomyces*, *Bacteroides*, *Fusobacterium* etc.^8,9^

Multiple factors sustain the triumph of the endodontic treatment, with the elimination of microbes being the most cardinal one.^10^ However, to achieve this, precise recognition of microbes is crucial to understand the pathogenesis of pulpal and periradicular diseases and to provide effective antimicrobial irrigation and medicament.^7^

Thus the current study aimed to assess and report the prevalence of facultative anaerobes from the root canals of deciduous molars.

## Methods

The present study was a prospective, randomized clinical trial on patients presenting for routine endodontic therapy in the Department of Pedodontics and Preventive Dentistry and the ethical clearance for the same was obtained from institutional Ethics Committee. Sixty cases of deciduous molars with necrotic pulp were selected based on the inclusion criteria.

### Inclusion criteria

- Age group: 6‒9-year-old children
- Children with no systemic and medical conditions
- Children not on any antiinflammatory drugs/antibiotics at least for 3 months

#### Clinical findings

- Multi-rooted teeth with adequate coronal structure for proper isolation, temporization and restoration
- Multi-rooted tooth with pulp involvement

#### Radiographic findings

- Radiolucency involving enamel, dentin and pulp in multi-rooted teeth
- Multi-rooted teeth with furcation involvement
- Multi-rooted teeth with periapical rarefaction with any of the roots
- Teeth having at least 2/3 of root length

After recording the case history and obtaining informed consent, local anesthesia was administered and the offending tooth was isolated with a rubber dam. The endodontic field was swabbed with povidone iodine to eliminate surface contaminants for 3 minutes and then rinsed with sterile saline solution; then 5% sodium thiosulphate solution was used to inactivate the iodine tincture so that its remnants would not hinder microbial sampling.

Following disinfection protocol, an access opening was made using a round bur at high speed; deroofing was carried out and the coronal pulp was removed. The canals were located and the pulp tissue was extirpated using barbed broach, following which the canal length was determined by the conventional Ingle’s radiographic method. The samples were obtained from the palatal and distal canals of maxillary and mandibular molars, respectinely.^11^ The contents of the canal were absorbed into sterile paper points at the most apical extent of the canal for 60 seconds; the saturated paper points were deposited into 10 mL of thioglycollate broth for anaerobic culture. Chemomechanical preparation was carried out using stainless steel H–files up to #30/35; the root canals were dried using absorbent paper points and obturated with zinc oxide-eugenol by means of hand pluggers to push the paste just short of the apex. The coronal space was filled with type II GIC cement.

### Microbiological processing

The samples (paper points) placed in 10 mL of thioglycollate for anaerobic culture were vortexed in a vortex mixer (Eltek VM 301) for 1 minute. Sample dilutions of 10, 100, 1000, 10000, 100000-fold were prepared. Serial dilution was carried out as follows.

One mL of transport medium containing the sample was transferred to the vial containing 9 mL of sterile peptone water bringing in 10^-2^ dilution. Then, 1 mL from this vial was added to the next vial containing 9 mL of sterile peptone water to make it a 10^-3^ dilution. This procedure continued in a similar manner up to 10^-5^ dilution.

Lab procedures were conducted according to CLSI (Clinical and Laboratory Standard Institute) guidelines. The nutrient agar plates were inoculated with 0.1 mL of undiluted sample (10 dilution) as well as each of the four dilutions with the help of sterile spreaders. For anaerobic samples, nutrient agar plates were kept in GasPak jar and incubated for up to 48 hours. The growth was observed in each medium after respective incubation. The purity of cultures was assessed by employing gram staining, observing the morphology of colonies on blood agar plates and using a biochemical identification kit: *E. coli* by IMViC test, *E. faecalis* by bile esculin test, *S. mutans* by esculin test and catalase test, *Staphylococcus aureus* by catalase and coagulase test. The data thus obtained were analyzed per canal in terms of the mean counts and frequency of each microorganism.

## Results

*Enterococcus faecalis* was isolated in 30% (18 samples) of cases, *Escherichia coli* in 28.4% (17 samples), *Staphylococcus aureus* in 25% (15 samples), α-hemolytic *Streptococci* in 15% (9 samples) and *Proteus mirabilis* in 1.6% (1 sample) of cases.

**Table 1:** Prevalence of facultative anaerobes from the root canals of deciduous molars

Sample no	Organism	colony count (cfu/ml)
1	*Staphalococcus aureus*	6.7 × 10^4^
2	*Staphalococcus aureus*	5.3 × 10^4^
3	*Escherichia coli*	9.3 × 10^4^
4	*Staphalococcus aureus*	120 ×10^4^
5	*Staphalococcus aureus*	7.9 ×10^4^
6	*Staphalococcus aureus*	8.4 ×10^4^
7	*Enterococcus faecalis*	6.2× 10^4^
8	*Enterococcus faecalis*	9.3× 10^4^
9	*Staphalococcus aureus*	210× 10^4^
10	*Alpha Haemolytic Streptococci*	6.9× 10 ^4^
11	*Enterococcus faecalis*	6.8× 10^4^
12	*Escherichia coli*	5.4 × 10^4^
13	*Enterococcus faecalis*	6.3 × 10^4^
14	*Enterococcus faecalis*	7.4 × 10^4^
15	*Escherichia coli*	3.4 × 10^4^
16	*Alpha Haemolytic Streptococci*	2.1 × 10^4^
17	*Escherichia coli*	3.6 × 10^4^
18	*Enterococcus faecalis*	2.4 × 10^4^
19	*Escherichia coli*	3.1 × 10^4^
20	*Enterococcus faecalis*	5.4 × 10^4^
21	*Escherichia coli*	4.3 × 10^4^
22	*Staphalococcus aureus*	3.1 × 10^4^
23	*Enterococcus faecalis*	2.9 × 10^4^
24	*Escherichia coli*	3.1 × 10^4^
25	*Escherichia coli*	5.1 × 10^4^
26	*Staphalococcus aureus*	6.1 × 10^4^
27	*Escherichia coli*	4.2 × 10^4^
28	*Staphalococcus aureus*	2.9 ×10^4^
29	*Enterococcus faecalis*	4.2 × 10^4^
30	*Escherichia coli*	5.7 × 10^4^
31	*Staphalococcus aureus*	4.2× 10 ^4^
32	*Staphalococcus aureus*	105× 10 ^4^
33	*Alpha Haemolytic Streptococci*	6.2× 10 ^4^
34	*Alpha Haemolytic Streptococci*	6.8× 10^4^
35	*Escherichia coli*	5.2× 10^4^
36	*Alpha Haemolytic Streptococci*	3.6× 10^4^
37	*Enterococcus Faecalis*	7.2× 10^4^
38	*Escherichia coli*	6.4× 10^4^
39	*Enterococcus Faecalis*	7.5× 10^4^
40	*Staphalococcus aureus*	81× 10^4^
41	*Alpha Haemolytic Streptococci*	8.7× 10 ^4^
42	*Escherichia coli*	5.9× 10^4^
43	*Staphalococcus aureus*	6.1× 10 ^4^
44	*Enterococcus Faecalis*	7.4× 10^4^
45	*Proteus Mirabilis*	5.9× 10 ^4^
46	*Enterococcus faecalis*	4.2× 10^4^
47	*Enterococcus faecalis*	7.2× 10^4^
48	*Staphalococcus aureus*	26× 10^4^
49	*Alpha Haemolytic Streptococci*	3.4× 10^4^
50	*Enterococcus faecalis*	4.8× 10^4^
51	*Enterococcus faecalis*	4.3× 10 ^4^
52	*Escherichia coli*	2.9× 10^4^
53	*Enterococcus faecalis*	3.5 × 10^4^
54	*Escherichia coli*	2.9 × 10^4^
55	*Escherichia coli*	4.5 × 10^4^
56	*Alpha Haemolytic Streptococci*	3.4 × 10 ^4^
57	*Alpha Haemolytic Streptococci*	4.2 × 10^4^
58	*Staphalococcus aureus*	3.9× 10 ^4^
59	*Escherichia coli*	3.7 × 10 ^4^
60	*Enterococcus Faecalis*	2.8 × 10 ^4^

## Discussion

To achieve favorable prognosis following endodontic treatment, eradication of microbes is mandatory, but it is more exasperating in children due to complex root canal system and proximity to the permanent tooth bud, along with superadded difficulty in behavior management. ^1,2,3^

Development of pulp and periapical diseases is determined by many factors resulting in penetration of microbes into the root canal system. In 1894, W.D Miller was a pioneer to proclaim his observations regarding infected root canals. Since then, bacteria have been implicated in infections of endodontic origin.^12^

The fate of endodontic infection was revolutionized with the advent of strict anaerobic culture techniques as the rarely isolated anaerobic microorganisms were mainly isolated from infected root canals of permanent teeth.^4,5^

The polymicrobial nature of endodontic microflora has been well documented.^6^ Recurrent microorganisms that have been cultured from necrotic deciduous root canals include *Streptococcus salivarius*, *Hemolytic Streptococci* (α, β, γ), and *Enterococcus faecalis*.^13,14^ In the present study, several microorganisms were isolated, including *Enterococcus faecalis*, *Escherichia coli*, *Staphylococcus aureus* and α-hemolytic *Streptococci*, confirming the polymicrobial nature of colonization of root canals of deciduous molars.

Teeth with pulp necrosis and periapical involvement with at least one root were selected as these teeth have a poor prognosis compared to teeth without periapical pathology.^15^

The tooth surface was disinfected using 5% povidone iodine ‒ a simple effective method shown to be efficacious and widely used.^16^

Grossman^17^ advocated the use of sterile paper points for obtaining the root canal samples and advocated that 1‒10 microorganisms are optimum for obtaining growth through culture procedures. However, Molander et al^8^ pointed out the dissemination of microbes in each and every part of root canals of deciduous teeth such as the lumen, dentinal tubules, accessory canals, periapical biofilm, apical delta and apical foramen. Considering both these aspects, microbial samples were collected from the most difficult area to be cleaned, i.e. the apical third of the canal. Adsorbent paper points were used for collection of microbiological samples as they collect bacteria present only inside the root canal and not from the dentinal tubules or deeper areas, thus reducing the chances of variability in quantity and type of microbial isolation from the root canals.

In the current study, *Enterococcus faecalis* was primarily isolated in 30% of cases. *Enterococcus faecalis* has been reported in 10‒30% of root canal failures, inflamed root canals and in untreated canals; it constitutes about 5% of the total microflora.^19,20^This is attributed to the ability of *Enterococcus* to persist in extreme environments of root canals, which includes high pH and salt concentrations. Also it is fortified with serine protease, gelatinase and collagen-binding protein (Ace) which helps in the attachment of *E. faecalis* to dentin. *E. faecalis* is fortified to survive extended spans of starvation. In addition, it can form adherent biofilms on the root canal walls, which eventually undergo calcification within a period of 6 weeks, making its eradication from the root canals extremely difficult.^14^

It has been reported that *E. coli* is mainly isolated from persistent periapical infections and its endotoxin plays a key role in exacerbating the infectious state.^19,21^ In the present study, it was isolated in 30% of cases. Also a study conducted to evaluate the prevalence of microbes in the root canals of deciduous teeth diagnosed with irreversible pulpitis isolated *E. coli* in about 15% of cases.^22^

*Staphylococcus aureus* is a gram-positive facultative anaerobe with the ability to remain viable for extended periods as it is resistant to drying and temperature changes. In the present study it was isolated from 25% of cases. Hegde and Pallavi^22^ and Cohen et al^23^reported the presence of *Staphylococcus aureus* in 5% and 67% of cases, respectively.

Similarly, α-hemolytic *Streptococci* were found in 15% of cases whereas studies carried out by Pazelli et al^1^and Hegde and Pallavi^22^ revealed the presence of *Streptococci* in 96.7% and 100% of cases, respectively. Presence of these opportunistic microorganisms may vary from person to person, depending on individual’s oral hygiene.

In addition,, in a single case the presence of *Proteus mirabilis* was documented. To date literature search has not revealed isolation of this microorganism from infected root canals of deciduous molars.^24^

There are limitations to this study, showing the need for further studies involving substantial sample size along with the combination of PCR and bacterial CFU counts to obtain better confirmation of true bacterial populations.

## Conclusion

Under the limitations of the present study, it can be concluded that the root canals of deciduous molars had a predominance of facultative anaerobes, confirming the polymicrobial nature of microbiota of root canals of deciduous molars.

## Acknowledgments

None

## Authors’ contributions

The study was designed and performed by the first author and the manuscript was drafted by both the authors.

## Funding

Study was self funded and was not funded by any institution or organization.

## Competing interests

The authors declare no competing interests with regards to the authorship and/or publication of this article.

## Ethics approval

The study was approved by the Institutional Ethics Committee of Swargiya Dadasaheb Kalmegh Smruti Dental College and Hospital, Nagpur, Maharashtra, India.
